# Acute oxygenator occlusion in two cases of polycythemia vera: Bailout strategies

**DOI:** 10.1111/jocs.14876

**Published:** 2020-07-15

**Authors:** Philipp Kaiser, Andreas Zuckermann, Johann Horvat, Franz Lederer, Heinz Gisslinger, Franz Gremmel, Paul Simon, Dominik Wiedemann, Martin Andreas

**Affiliations:** ^1^ Department of Cardiac Surgery Medical University of Vienna Vienna Austria; ^2^ Department of Internal Medicine I Medical University of Vienna Vienna Austria

**Keywords:** myeloproliferative disorders, oxygenator clotting, polycythemia vera

## Abstract

Polycytemia vera (PV) is a rare myeloproliferative neoplasm associated with microcirculatory disturbances, thrombosis and bleeding. Patients suffering from PV have a high risk of perioperative adverse events, but the literature regarding on‐pump procedures in PV patients is scarce. We report two cases of acute and severe oxygenator failure during cardiopulmonary bypass and present valid exit scenarios.

## 1 INTRODUCTION

Polycytemia vera (PV) is a rare myeloproliferative neoplasm which may, mostly late in the course of disease, transform into myeloid metaplasia with myelofibrosis or into leukemia.^1^ Patient frequently report about microcirculatory disturbances, sometimes with erythromelalgia. Pruritus, and fatigue are also frequent symptoms and clinical investigation can reveal splenomegaly. Total red blood cell mass is significantly increased. Cell proliferation is typically independent of erythropoietin and a result of a mutation of JAK2 in hematopoietic progenitor cells.^2^ While therapy is usually applied to reduce long‐term complications, patients may have an increased cardiac risk profile including thrombosis and bleeding.^1^, ^3^ Phlebotomy or treatment with the chemotherapeutic agent hydroxyurea may be necessary to control hematocrit, white blood cell counts and platelet counts.^1^ Furthermore, antiaggregative and antithrombotic therapy may be required.

Patients suffering from PV have a high risk of perioperative adverse events.^4^ While several reports are available for noncardiac surgical procedures, the literature regarding on‐pump procedures is scarce.^5^ Cardiopulmonary bypass represents a severe change of hemodynamic conditions and specifically the oxygenator imposes high mechanical stress to the blood components. New generations of oxygenators have a very low risk of thrombosis due to their specific internal coating.^5^ However, this may not be sufficient in patients with PV. We report two cases of acute and severe oxygenator failure and present valid exit scenarios.

## 2 CASE 1

**Figure 1:**
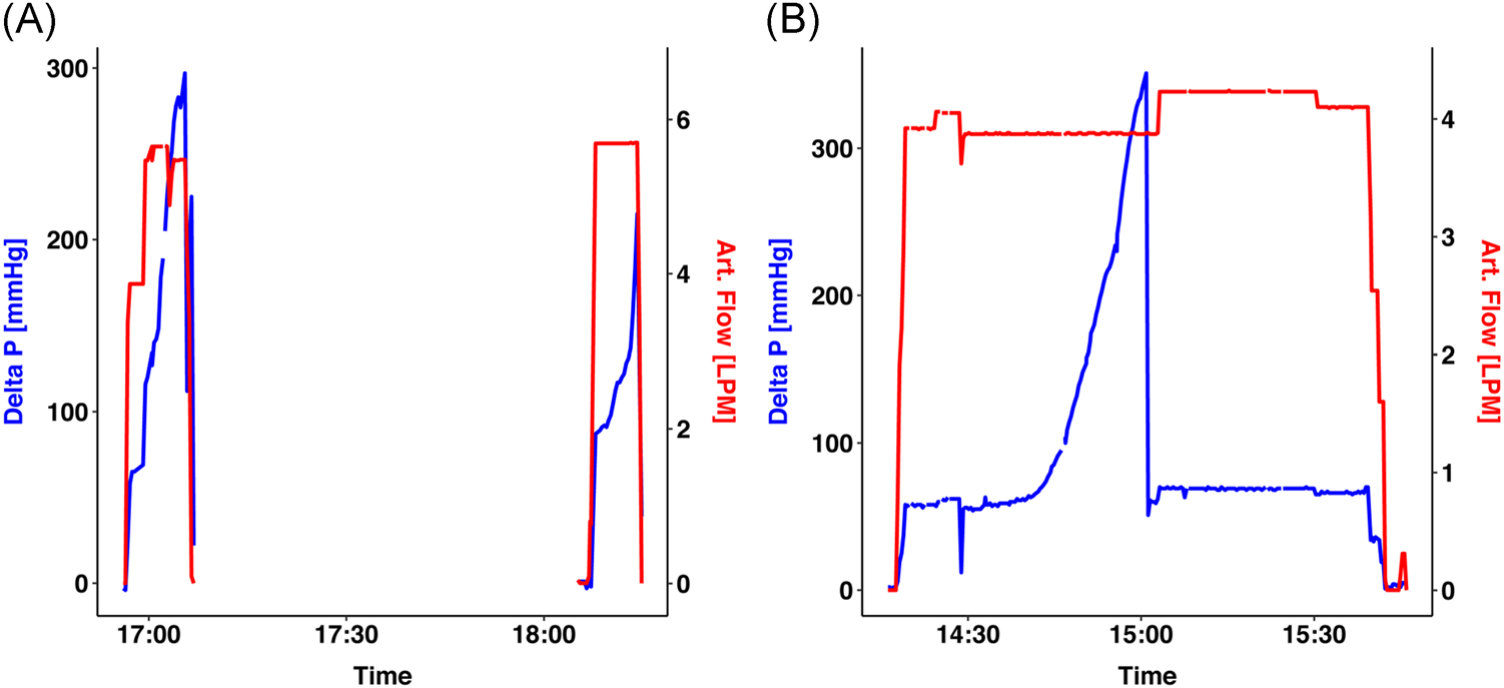
Perioperative pump flow (red) and oxygenator pressure gradient (blue). A, The first case revealed a rapid pressure gradient increase and was exchanges with a full pump stop, and a second clotting was observed. B, The second case had a back‐up oxygenator, and the change could be performed without pump stop during cardiac surgery

**Figure 2:**
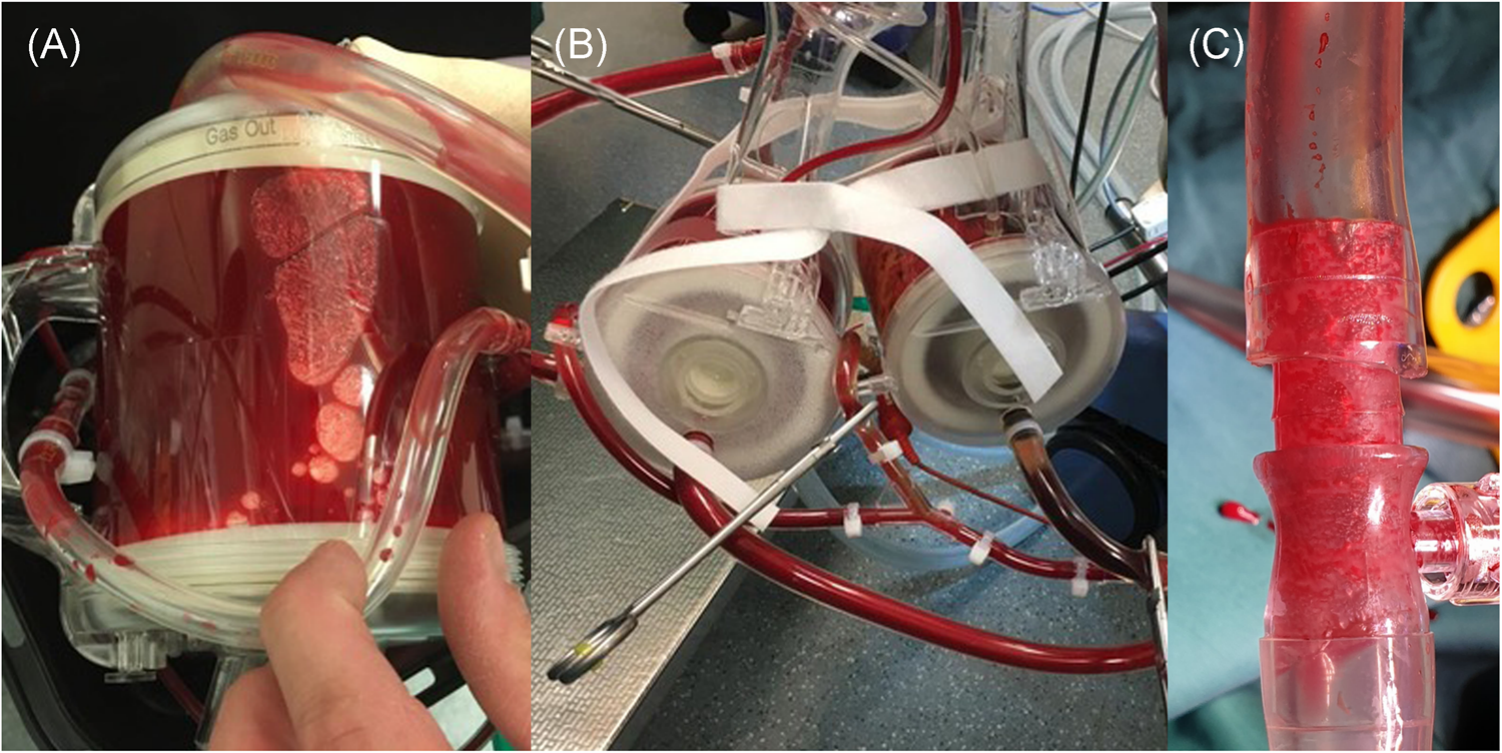
Intraoperative images. A, Clotted oxygenator (case 1). B, Back‐up oxygenator (case 2). C, Thrombotic adhesions found in pump tubes after surgery (case 2)

A 63‐year‐old patient with severe aortic stenosis (mean gradient 40mm Hg), reduced ejection fraction (37%) and worsening dyspnea NYHA III was admitted for surgical aortic valve replacement and tricuspid valve repair. He was suffering from PV and had a history of two pulmonary embolisms. The heart‐team decided for open surgical aortic valve replacement and tricuspid valve repair. The preoperative blood draw revealed an increased red blood cell count (hematocrit 54%), an increased white cell count (17 000/μL) and an increased number of platelets (980 000/μL). Initial activated clotting time (ACT) was 139 seconds, which increased to 1249 seconds after administration of 50 000 IE heparin and 2000 mg of tranexamic acid. The patient was taken on pump, but the oxygenator clotted after 12 minutes, luckily before aortic cross clamp (Figure 1A and Figure 2A). Thereafter, hemodilution was initiated with a total blood draw of 1550 mL and the oxygenator was changed. Volume was replaced with Ringer's lactate and plasma‐expander (500 mL). A second pump run was initiated 1 hour after the first pump run, but had to be stopped 7 minutes later due to repeated clotting. ACT was 846 seconds at this time. Surgery was aborted and the patient was closed. He recovered uneventfully and was scheduled for transcatheter aortic valve replacement (TAVR), which was successfully performed 6 days after the initial surgery. The preprocedural hematocrit was 32%. No specific measures were taken and the patient underwent transfemoral TAVR with a self‐expanding EvolutR 34 mm under conscious sedation. He was transferred to the intensive care unit thereafter to allow optimal monitoring, recovered uneventful and was discharged home at the third postoperative day in good clinical condition. The postoperative echocardiography showed excellent valve function without paravalvular leakage or stenosis and a reduced tricuspid regurgitation.

## 3 CASE 2

A 64‐year‐old female patient with severe aortic stenosis and severe mitral regurgitation was admitted for planned combined valve surgery. She was suffering from PV with a preoperative cell count of 4.7 × 10^6^/μL for red blood cell, 570 000/μL for thrombocytes and 11 500/μL for white blood cell. Current preoperative treatment for PV was ruxolitinib 5 mg in the morning and Ropeginterferon alfa‐2b 250 mg twice per week. Due to the institutional experience with PV in previous cases, a back‐up oxygenator was cut into the pump system before initiation of cardiopulmonary bypass (Figure 2B). Total 500 mL of the patients own blood was drawn before normothermic cardiopulmonary bypass for hemodilution and re‐infused after surgery. Preoperative ACT was 126 seconds, which increased to 512 seconds after 35 000 IE of heparin. However, ACT fell rapidly to 245 seconds after 25 minutes and a repeated dose of 15 000 IE was given. Tranexamic acid (1000 mg) was additionally administered before cardiopulmonary bypass. While a 27 mm Medtronic Mosaic biological valve was implanted, the oxygenator pressure increased and the system was switched to the back‐up oxygenator (Figure 1B). The surgery was continued by replacing the aortic valve with a rapid‐deployment surgical bioprosthesis (Edwards Intuity Elite 19 mm). The surgery remained uneventful and the patient could be weaned from bypass without adverse events. Pump tubing showed severe thrombotic adhesions at the connection areas (Figure 2C). Further postoperative course remained uneventful and the patient was discharged in good clinical condition.

## 4 COMMENT

PV was previously identified as a risk factor during noncardiac surgery.^4^ Furthermore, acute intracardiac thrombosis was reported during coronary artery bypass grafting.^6^, ^7^

Both patients presented herein are defined as PV patients with a high risk for thrombosis according to previous literature.^8^ Fieldwalker et al^5^ previously reported a case with a sudden and very high increase of trans‐oxygenator gradients, impairing pump flow and requiring intensive measures to improve pump flow. These group was not able to change the oxygenator during surgery due to the hemodynamic condition of the patient, but were able to improve the gradient with further hemodilution and intensified antiaggregation. Interestingly, this patient's flow improved over time and surgery could be concluded.

We report herein two additional PV patients with severe adverse events related to the oxygenator. The first patient had two episodes of rapid oxygenator closure, even after significant hemodilution and anticoagulation. Therefore, this patient was switched to a transfemoral approach, which was uneventful. The second patient required a combined aortic and mitral procedure and was therefore planned for surgery instead of a transfemoral approach. Due to our previous experience with PV during cardiopulmonary bypass, a back‐up oxygenator was prepared and saved this patient from a severe adverse event during cardiopulmonary bypass.

We want to highlight the increased risk of patients suffering from PV and undergoing cardiac surgery. This risk seems to be not directly related to the actual blood cell count, but may also be induced by cell dysfunctions with increased clotting capability in these patients. Hemodilution did not prevent a second oxygenator clotting in our experience. Currently, no verified method exists to predict clotting of the oxygenator in patients with PV. Furthermore, patients with PV may also present with an increased bleeding risk in the postoperative period.^9^, ^10^ Therefore, we recommend to apply transcatheter strategies when a comparable outcome can be expected. If cardiopulmonary bypass is required, a back‐up oxygenator should be included in the pump system to be ready for rapid exchange.

## DISCLOSURES

M. Andreas is proctor (Abbott, Edwards) and advisor (Medtronic). G. Laufer is proctor (Edwards).

## ETHICS STATEMENT

The patients gave written informed consent to publish their cases.
